# Modeling Possible Cooling-Water Intake System Impacts on Ohio River Fish Populations

**DOI:** 10.1100/tsw.2002.167

**Published:** 2002-04-26

**Authors:** Elgin Perry, Greg Seegert, Joe Vondruska, Timothy Lohner, Randy Lewis

**Affiliations:** Consulting statistician, 2000 Kings Landing Rd., Huntington, MD 20639, USA; EA Engineering, Science and Technology, Deerfield, IL 60015, USA; American Electric Power, Columbus, OH 43215, USA; Cinergy, Plainfield, IN 46168-1782, USA

**Keywords:** Clean Water Act 316(b), entrainment, fish, impingement, population modeling, Ohio River

## Abstract

To assess the possible impacts caused by cooling-water intake system entrainment and impingement losses, populations of six target fish species near power plants on the Ohio River were modeled. A Leslie matrix model was constructed to allow an evaluation of bluegill, freshwater drum, emerald shiner, gizzard shad, sauger, and white bass populations within five river pools. Site-specific information on fish abundance and length-frequency distribution was obtained from long-term Ohio River Ecological Research Program and Ohio River Sanitation Commission (ORSANCO) electrofishing monitoring programs. Entrainment and impingement data were obtained from 316(b) demonstrations previously completed at eight Ohio River power plants. The model was first run under a scenario representative of current conditions, which included fish losses due to entrainment and impingement. The model was then rerun with these losses added back into the populations, representative of what would happen if all entrainment and impingement losses were eliminated. The model was run to represent a 50-year time period, which is a typical life span for an Ohio River coal-fired power plant. Percent changes between populations modeled with and without entrainment and impingement losses in each pool were compared to the mean interannual coefficient of variation (CV), a measure of normal fish population variability. In 6 of the 22 scenarios of fish species and river pools that were evaluated (6 species × 5 river pools, minus 8 species/river pool combinations that could not be evaluated due to insufficient fish data), the projected fish population change was greater than the expected variability of the existing fish population, indicating a possible adverse environmental impact. Given the number of other variables affecting fish populations and the conservative modeling approach, which assumed 100% mortality for all entrained fish and eggs, it was concluded that the likelihood of impact was by no means assured, even in these six cases. It was concluded that in most cases, current entrainment and impingement losses at six Ohio River power plants have little or no effect at the population level.

### INTRODUCTION

Cooling-water intake systems have the potential to adversely impact aquatic organisms through entrainment and impingement. Entrainment occurs when organisms (e.g., larval fish) pass through the intake traveling screens and into the power plant where they may suffer injury or death. Impingement occurs when organisms are drawn against intake trash racks or screens by the force of the incoming water current.

Section 316(b) of the Clean Water Act (CWA) requires that “the location, design, construction and capacity of cooling water intake structures reflect the best technology available for minimizing adverse environmental impact.” Historically, the EPA has allowed section 316(b) of the Clean Water Act to be evaluated on a case-by-case basis, with individual plants performing 316(b) "demonstrations." These 316(b) demonstrations consisted of quantifying entrainment and impingement rates, then assessing whether the measured rates would affect populations of at-risk species. This has resulted in considerable variation in compliance requirements from plant to plant.

A key issue in the 316(b) rule development process has been how to define "adverse environmental impact" (AEI). The loss of a single fish could be considered an adverse impact and lead to an in-depth analysis of the number of fish killed and the cost of installing new intake technologies. However, the electric utility industry does not believe that Section 316(b) of the CWA was intended to address the loss of individual fish, but instead, was written to address the potential adverse impact on fish populations. The fact that an individual fish may die or suffer adverse physiological changes does not imply that the population will suffer a harmful decrease in number. In fact, the results of long-term monitoring studies in the Ohio River through 1985 have demonstrated that, within the permanent restrictions placed upon the river ecology by the lock and dam system, there is strong evidence of positive changes in the fish community due to improvements in water quality[1]. This has occurred in spite of the loss of millions of fish due to entrainment and impingement.

It is not possible to simply measure entrainment and impingement at a power plant and directly relate the results to population-level impacts. Rather, a suggested first step in assessing potential AEIs is to assess the condition of affected populations and to model the impact of various entrainment and impingement scenarios on those populations. This has the advantage of avoiding unnecessary studies and helping to focus actual field studies on those populations that are most vulnerable to adverse impact. Therefore, to assess whether Ohio River power plants may be adversely affecting fish populations, a Leslie matrix model was developed. For each fish species chosen, several life history parameters (age, growth, fecundity, and age-specific survival) were used and the population was projected forward for a specified time period (50 years). From the 316(b) perspective, the advantage of this approach is that it can be used to model the population as it currently is (i.e., with the power plant operational, inclusive of plant-specific entrainment and impingement losses) and it can be used to model the population assuming these losses were not occurring. By comparing populations with and without these losses, it can be judged whether the losses are of sufficient magnitude to significantly affect population size.

### METHODS

#### Formulating the Population Model

Matrix models are widely used in ecology to investigate the structure and dynamics of natural populations[2,3,4,5]. The advantage of matrix models over other population models such as the logistic model, Ricker's spawner-recruit model, or the Beverton-Holt formulation, is that matrix models prescribe differing vital rates for different parts of the population while other models treat all individuals in the population as if they are identical. Because length data are available from the Ohio River electrofishing studies, it seems appropriate to partition the population into length categories and allow for the possibility that survival and fecundity rates may differ among size classes. One criticism of matrix models is that for populations with continuous reproduction, matrix models are not well suited. Matrix models use a time step that assumes that all births occur at the beginning of the time interval. For fish populations that typically have a relatively short spawning season, this assumption of a pulse of reproduction at the beginning of the time interval works well.

The basic form of the Leslie matrix model is described by Leslie[6,7]:

$$\begin{pmatrix} f_1 & f_2 & \cdots & f_k \\ s_1 & 0 & \cdots & 0 \\ . & . & \cdots & . \\ . & . & \cdots & . \\ 0 & 0 & s_{k-1} & 0 \end{pmatrix} \begin{pmatrix} n_1 \\ n_2 \\ . \\ . \\ n_k \end{pmatrix}_t = \begin{pmatrix} n_1 \\ n_2 \\ . \\ . \\ n_k \end{pmatrix}_{t+1} \quad (1)$$

where $s_i$ is the probability that an individual will advance to the next age class, $f_i$ is the recruitment rate for age class $i$, $n_i$ is the number of individuals in size class $i$, $t$ is the ordinal for time step (year), and $k$ is the maximum number of size classes.

In its basic form, the Leslie matrix model is deterministic. That is, given one set of estimates for the parameters on one initial population vector, it will always predict the same population trajectory. The current implementation of the Leslie matrix model uses the estimates described below as the mean of stochastic distributions to simulate random year-by-year variability in the $s_i$ and $f_i$ vital rates. The details of this simulation are as follows.

With each annual projection of the population, survival parameters ($s_1$ through $s_k$) were simulated from a Beta distribution with the range of the random number generator limited to the interval (0, 1). The means of these survival estimates were set equal to the survival estimates obtained from a length-frequency model. The variance of these survival estimates was restrained so that the coefficient of variation (CV) was 25% for survival in the interval (0.10 to 0.90) and 10% for survival outside this interval.

Only the fecundity component of the recruitment estimates was randomly simulated. The sex ratio, proportion of mature females, and larval survival components were held constant. The fecundity component was simulated using a Poisson distribution with the mean determined from fecundity estimates taken from literature values[8,9,10,11,12]. As described below, the larval survival parameter was tuned to yield long-term stability.

There are numerous strategies in the modeling literature for implementing compensation, such as the Ricker spawner-recruit model and the Beverton-Holt equations. In this model, compensation is implemented by the simple idea that each pool in the Ohio River has a carrying capacity for each age class of each species. That is to say, the population may be controlled by the population vital rates up to this threshold, but then some external factor, such as food supply, nesting sites, or habitat, limits the population. The carrying capacity for each age class was set equal to the maximum abundance observed for that age class for the period of record. If during simulations, the projected age-specific abundance exceeded the carrying capacity for the age class, the age-specific abundance ($n_i$) was set equal to the carrying capacity.

### Data Sources

Since 1970, fish data have been collected near several power plants on the upper and middle Ohio River as part of the Ohio River Ecological Research Program (ORERP)[13]. Over this 30-year period, data have been collected primarily near six power plant locations:

| Plant | River Mile | Pool | River Miles |
|---|---|---|---|
| WH Sammis | 53.9 | New Cumberland | 31.7–54.4 |
| Cardinal | 76.7 | Pike Island | 54.4–84.4 |
| Kyger Creek | 260.0 | Robert Byrd | 237.5–279.2 |
| Beckjord | 453.0 | Markland | 436.2–531.5 |
| Tanners Creek | 494.0 | Markland | 436.2–531.5 |
| Clifty Creek | 560.0 | McAlpine | 531.5–605.0 |

While the ORERP was designed to gather information on the potential impacts of power plant operation on Ohio River biota, it did not require the collection of specific information on fecundity, recruitment, survival, or age and growth for individual Ohio River species[14]. This information was therefore obtained from the literature as noted in the following discussions. Only electrofishing data were used for the modeling effort as electrofishing is the single most effective sampling gear[15], and it has been used consistently as part of the ORERP. Since 1991, electrofishing has been conducted at night, rather than during the day, to be consistent with the approach recommended by Ohio EPA. Because catch rates and species composition changed in response to this change in sampling protocol, only electrofishing data from 1991 to 1998 were used in the model. Nighttime electrofishing data from 1992 to 1998 collected by ORSANCO were also used to supplement the ORERP electrofishing data.

Losses due to impingement and entrainment were based on data extracted from 316(b) study reports prepared for the above-listed power plants and for the Philip Sporn and Miami Fort power plants located in the Robert Byrd and Markland pools, respectively. Even though survival for some entrained species can be relatively high, the model conservatively assumed that all entrained fish were killed. Likewise, it was assumed that all impinged fish were killed.

### Selecting Species and Estimating Vital Rates

Species were selected based on the availability of sufficient data to describe the population characteristics of that species, the availability of related data (e.g., fecundity), and the vulnerability of each species to entrainment and impingement. Based on the above criteria, six species were selected: bluegill, freshwater drum, emerald shiner, gizzard shad, sauger, and white bass. For three of these species (bluegill, gizzard shad, and sauger) sufficient data were available to allow populations to be estimated for all five pools studied, while data on other species (e.g., emerald shiner) restricted the analyses to as little as one pool. The Leslie matrix model used to estimate the Ohio River fish populations requires estimates of survival and fecundity. Fecundity estimates for each species were taken from relevant fisheries' literature[8,9,10,11,12]. These estimates were converted to recruitment estimates by estimating survival to midsummer for young-of-the-year (YOY) fish as described under the Recruitment Estimates section (below). Survival estimates between sequential age classes were obtained from length-frequency data using the length-frequency model also described below. Several intermediate steps were undertaken to obtain the length-frequency model, including estimation of a growth curve, adjusting the data for size-dependent electrofishing capture probability, and adjusting the data for within-season growth.

### *Recruitment Estimates*

One major component of the Leslie matrix model is the recruitment component, which we define as the process of getting from eggs per female to YOY in midsummer after spawning. This is the component about which there exists the least amount of site-specific information. To obtain a reasonable estimate of the recruitment that is generated in each year, age-specific estimates of the number of eggs spawned per sexually mature female were compiled from the literature[8,9,10,11,12]. We assumed a sex ratio of 50:50. These estimates were used to set the mean of the stochastic distribution of the number of eggs spawned for the model. To obtain recruitment, the number of these eggs that survive to become YOY fish must be estimated. To model this survival, a parameter called larval survival (ls) was included in the model. While we call this the larval survival factor, it is the term that models the life history parameters: fraction of females that are mature and fraction of eggs that are actually spawned. Lacking information on all of these life history processes between number of eggs and YOY, larval survival was obtained by tuning the model to stability. Larval survival was set to the lowest level that would produce long-term stability for the population with the power plants operating. Stability was ascertained based on no observable increase or decrease in a graphical display of a 50-year projection using the stochastic version of the model. The rationale for this choice is that if the species continues to persist in the presence of the power plants, it must have achieved at least this minimal level of stability. Setting the parameter to this minimum level maximizes the effect of removing the power plants' influence on the long-term population level.

The data suggest that most species periodically have a good recruitment year. The model was designed so that approximately one year in five (randomly determined) is selected to be a good recruitment year in which YOY survival is ten times better than in a typical year. The parameter influencing recruitment is the high recruitment (HR) parameter. A Bernoulli random variable which takes on values of 1 or 0, was simulated to have probability 1/5 of being equal to 1. The HR parameter was simulated once for each projection. If HR = 1, then the model estimates a high recruitment year by increasing larval survival (ls) by an order of magnitude (i.e., ls is replaced by ls × 10). The initial population vector was set equal to the mean population level for the period of record.

#### Growth Model

The von Bertalanffy growth equation[16,17] was used to model length as a function of age. This model follows the form:

$$l_a = l_{inf}\,(1 - e^{(-k\,*(a\,-\,a_0))}) \tag{2}$$

where $l_a$ is the length at age $a$, $l_{inf}$ is the maximum length, $k$ is a growth rate coefficient, and $a_0$ is the initial length at age 0, usually set to 0. Estimates for the parameters for this nonlinear model were obtained using the nonlinear regression (NLIN) procedure of the SAS system[18]. The von Bertalanffy growth model served two purposes in the development of the length-frequency model. It was used to adjust the lengths of fishes caught at various times during the growing season to a common point in time. It was also used to determine distance between year class modes in the length-frequency model.

#### Length Adjustments

During each year of the electrofishing survey, collections were made in the late spring, midsummer, and early fall. The ideal data for parameterizing a Leslie matrix model would be an intensive snapshot of the population at a fixed date every year. In order to compose a length-frequency distribution at one point during the year, the lengths of the fish were adjusted by the von Bertalanffy model to estimate the length each fish would have been in midsummer (July 19). Using mean growth data obtained from the literature, the parameters of the von Bertalanffy model were estimated. Assuming an 8-month growing season, fish lengths were adjusted to the expected length for July 19. Lengths of fish captured in late spring were increased, while those of fish captured in the fall were decreased. Fish that were measured individually were assigned an adjusted length and then assigned to appropriate length groups. Fish that were measured in groups were prorated into adjacent length groups in proportion to the size of the adjustment.

#### Electrofishing Efficiency Adjustment

Because electrofishing is known to have a lower efficiency for collecting smaller fish, adjustments for electrofishing efficiency were made using a capture probability curve estimated for brown trout[19]. To adjust the data, the frequency for each length interval was multiplied by the inverse of the capture probability for the midpoint of the interval.

### Length-Frequency (Mixed Beta) Model

The concept behind the methods employed to obtain estimates of survival rates is based on the idea that each age class of a species will have a length distribution. As the fish grow older the mean of the age specific length distribution will increase in a manner described by a growth function and the variance may also change. As fish age and approach the maximum size for the species in question, it is almost certain that age-specific distributions will overlap. By compositing the age-specific distributions, one can obtain the population (all ages) distribution of lengths. The length-frequency model employed in this study attempts to estimate the mean and variance of each age-specific length distribution by fitting the composite model to the population length-frequency histogram. After obtaining the parameters for the age-specific length distributions, it is then possible to compute survival estimates as the ratio of expected frequency at age $i + 1$ divided by the expected frequency at age i. The length-frequency model employed here was similar in structure to the length-frequency models used in the ELEFAN system[20]. In the Ohio River model, the length-frequency data are represented by a mixture of scaled beta density functions, whereas the ELEFAN system uses normal density functions. The classical beta density described in any mathematical statistics book is defined on a domain of {0, 1} and is defined as follows:

$$f(x;a,b)=\frac{x^{a-1}(1-x)^{b-1}}{B(a,b)} \quad 0<x<1, a>0, b<0$$
$$B(a,b)=\frac{\Gamma(a)\Gamma(b)}{\Gamma(a+b)} \qquad (3)$$

where $\Gamma(b)$ is a complete gamma function and *a* and *b* are shape parameters for the beta density[21]. In order to use the beta density to define the probability that a randomly selected fish of a certain age falls in a particular length interval, the variable *x* must be rescaled from the interval {0, 1} to the interval {*minimum*, *maximum*} where *minimum* and *maximum* are defined as the minimum and maximum length for each year class of fish.

### Survival Estimates

The conditional probability of survival from age class *i* to age class *i* + 1 is estimated by the ratio of the expected frequency for age class *i* + 1 to the expected frequency for age class *i*. The expected frequencies are determined from the length-frequency model. It is called the conditional probability because it is the probability of surviving to the next age class conditioned on having survived to the current age class. These conditional probabilities give us the survival terms of the Leslie matrix model. In the case where the population should have more age classes than are represented in the length-frequency data (as shown in the growth data found in the literature), the survival of additional age classes was set equal to that of the last class represented in the data.

#### *Mean Population and Carrying Capacity*

The mean age-structured population vector and the age-structured carrying capacity vector were respectively estimated by the mean and maximum of the age-structured population vectors for the period of record. After adjustments for capture probability and size, the mean number of fish per age class per hectare was computed assuming an effective shocking width of 4.25 m for the recorded length of the shocking effort. Multiple samples per year were averaged. If the data did not represent all the older age classes of the population vector for a species, these older age class vector components for the mean population vector and the maximum population vector were extrapolated based on the survival estimate obtained from the two oldest age classes represented in the data.

#### Power Plant Effects

Because the length-frequency data by which the model is calibrated were collected while the power plants were operating, the model up to this point estimates population behavior while under the influence of the power plants. To estimate the effects of entrainment and impingement, the model was modified to simply add back the entrainment and impingement harvesting. The total entrainment estimate for each pool is multiplied by larval survival (ls) and added to the YOY component of the population vector during each projection. Annual impingement estimates for each pool were summed by year class and these totals were added to the appropriate population cells in each projection of the population. In this configuration of the model, both the entrainment and the impingement effects are constants. The model projected the population 50 years into the future, and 500 simulations were run for both the power-plant-effect and no-power-plant-effect models.

### RESULTS

#### Growth Equation

The first step of the length-frequency modeling process is to obtain parameters for the von Bertalanffy growth equation. To develop this growth model, species-specific age and length data from the literature for the Ohio River or other appropriate water bodies were used. The example von Bertalanffy growth curves for sauger and gizzard

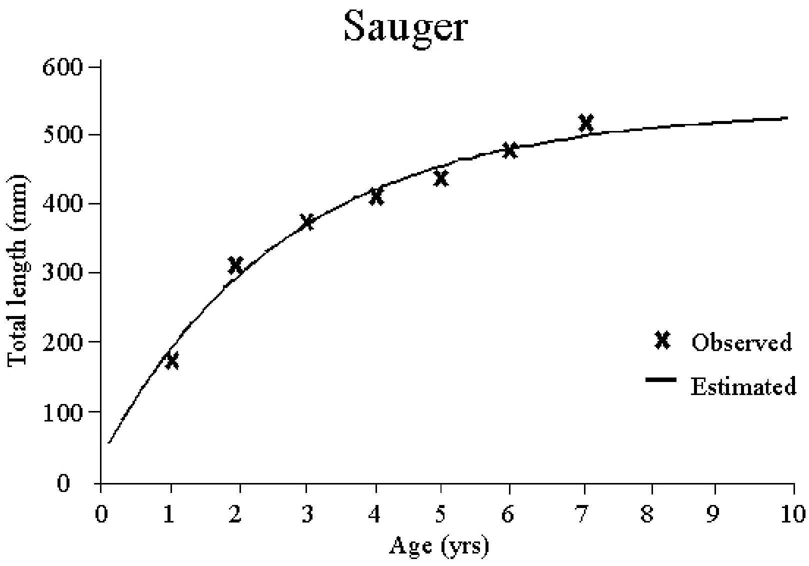

FIGURE 1A

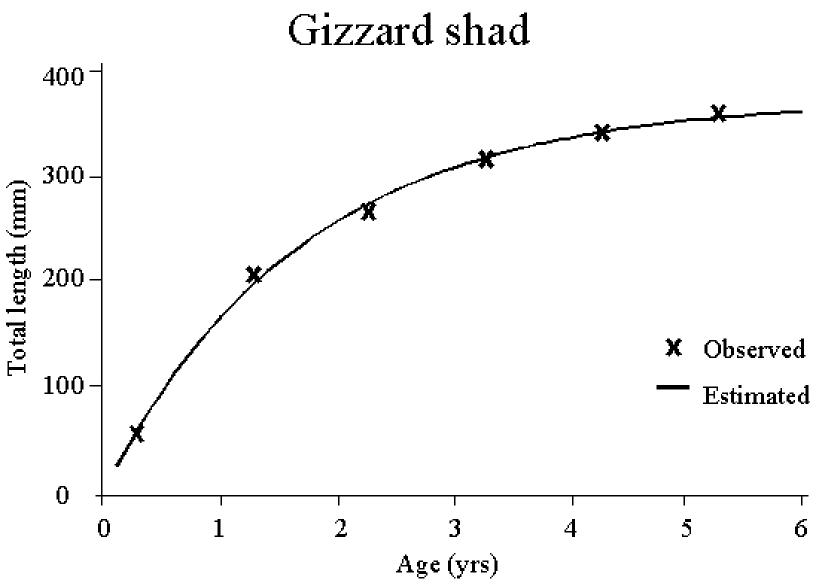

FIGURE 1B

**FIGURE 1.** Growth curves for sauger and gizzard shad showing observed data and estimated von Bertalanffy model.

shad (Fig. 1) and the parameters for the equations describing these curves for the remaining species (Table 1) indicated that the fit of the model to the actual data was acceptable. The lowest growth rates (K in Table 1) were obtained for emerald shiner and bluegill. The highest growth rate was obtained for gizzard shad, which is reasonable because gizzard shad is a fast-growing species. The LINF parameter in the von Bertalanffy model is a theoretical maximum length (at age infinity); however one would not expect fish to approach that length unless they were extremely long lived.

### Length-Frequency Model

Initially each species was modeled with as many age classes as were indicated in the growth data. When fitting the length-frequency model, it was common for the expected frequency of the last (youngest) age category to start converging toward zero. When this happened, the last age class would be eliminated under the

**TABLE 1**
**Statistics for the Growth Curves by Species**

| Species | Parameter | Asymptotic Estimate | Std. Error |
|---|---|---|---|
| Sauger | LINF | 533.3189072 | 34.335407684 |
| | K | 0.3711828 | 0.087560862 |
| | TO | -0.1473548 | 0.273224912 |
| Gizzard shad | LINF | 370.1974256 | 10.034100589 |
| | K | 0.5841744 | 0.054676357 |
| | TO | -0.0004830 | 0.051837130 |
| Freshwater drum | LINF | 458.0946917 | 12.177552434 |
| | K | 0.3298979 | 0.036129042 |
| | TO | 0.0071713 | 0.153225199 |
| White bass | LINF | 418.8457737 | 6.8258631136 |
| | K | 0.4490271 | 0.0375477085 |
| | TO | 0.0541263 | 0.1044621517 |
| Bluegill | LINF | 258.9146360 | 5.7377772322 |
| | K | 0.2568898 | 0.0198869626 |
| | TO | -0.6382159 | 0.1263056293 |
| Emerald shiner | LINF | 164.5188804 | 0.00000000000* |
| | K | 0.2460419 | 0.00068797160 |
| | TO | -0.2357573 | 0.00142707420 |

Note: LINF is the theoretical maximum length (mm) of a fish under the von Bertalanffy model; K is the growth rate parameter; TO is a parameter to adjust the age (year) of the initial cohort.

*With only 3-year classes, the von Bertalanffy model obtains a perfect fit which leaves no information for estimating error.

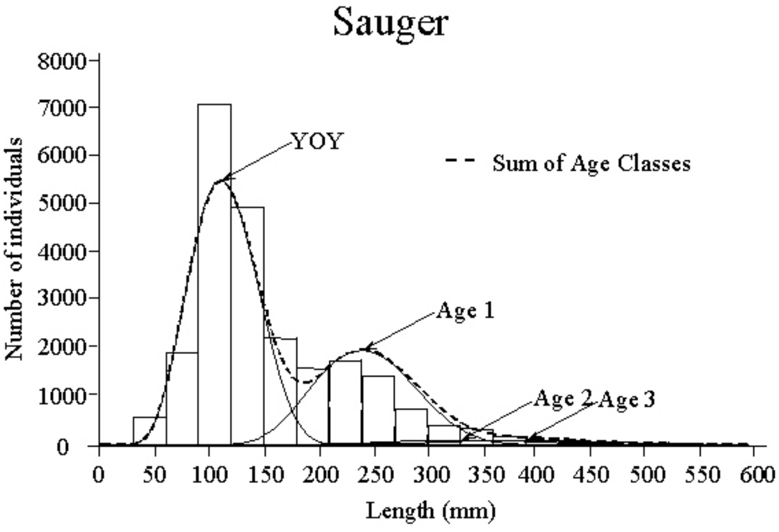

FIGURE 2A

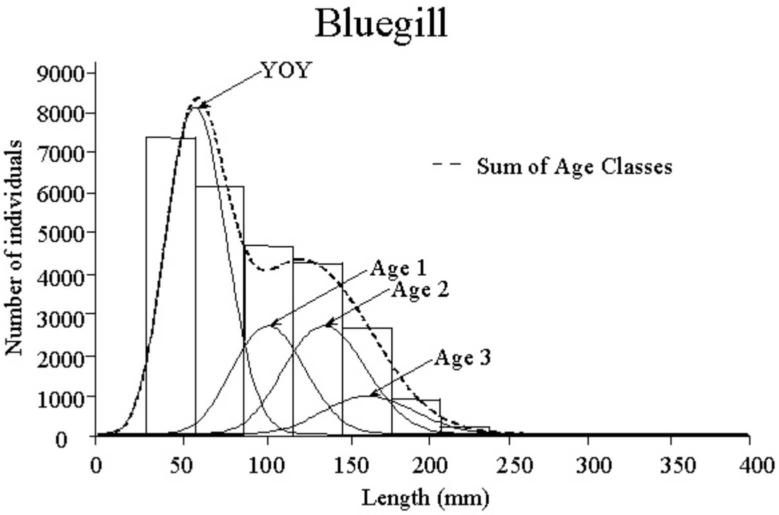

FIGURE 2B

**FIGURE 2.** The length frequency data (shown in 30-mm size groups) superimposed with the length frequency model for sauger and bluegill.

assumption that this age class (usually Age 0) was not adequately represented in the electrofishing data, and the fitting process was restarted with one fewer age category.

As a rule, the length-frequency model acceptably reproduced the observed length-frequency, as shown in the sauger example (Fig. 2A). One exception was the case of bluegill (Fig. 2B), where the second and third age classes (1- and 2-year-olds)

were fitted with almost equal weight, suggesting that there is very little mortality between age 1 and 2. This anomalous result is probably an artifact of the fact that 30-mm length categories are too broad for bluegill, a fairly slow-growing and rather small species. Categorizing the bluegill into 30-mm size groups resulted in too low a resolution to accurately define the peaks associated with these two age classes.

The mean length for each age class is expressed by the von Bertalanffy growth equation. The 25$^{th}$ and 75$^{th}$ percentiles of length for each age class were computed from the beta density function parameterized with $a$, $b$, and $m$ as defined previously (Table 2).

**TABLE 2**
**The Mean, 25$^{th}$ Percentile, and 75$^{th}$ Percentile Lengths (mm) for Each Modeled Species (by Age Class, as Estimated by the Length-Frequency Model)**

| | | Age | | | | |
|---|---|---|---|---|---|---|
| **Species** | **Statistic** | **0** | **1** | **2** | **3** | **4** |
| Sauger | 25$^{th}$ Percentile | 71.02 | 197.78 | 285.23 | 345.57 | 387.20 |
| | Mean | 90.34 | 227.70 | 322.47 | 387.85 | 432.96 |
| | 75$^{th}$ Percentile | 108.96 | 256.54 | 358.36 | 428.60 | 477.07 |
| Gizzard | 25$^{th}$ Percentile | 77.02 | 191.25 | 254.94 | 290.45 | 310.25 |
| shad | Mean | 93.77 | 216.07 | 284.26 | 322.28 | 343.48 |
| | 75$^{th}$ Percentile | 107.22 | 236.00 | 307.81 | 347.85 | 370.17 |
| Freshwater | 25$^{th}$ Percentile | 54.49 | 156.43 | 229.72 | 282.42 | 320.31 |
| drum | Mean | 69.66 | 178.81 | 257.29 | 313.72 | 354.29 |
| | 75$^{th}$ Percentile | 80.64 | 195.00 | 277.23 | 336.36 | 378.87 |
| White bass | 25$^{th}$ Percentile | 59.45 | 162.23 | 227.83 | 269.70 | 296.43 |
| | Mean | 84.23 | 205.28 | 282.54 | 331.85 | 363.32 |
| | 75$^{th}$ Percentile | 107.35 | 245.44 | 333.57 | 289.82 | 425.72 |
| Bluegill | 25$^{th}$ Percentile | 19.79 | 68.29 | 105.81 | 134.83 | 157.27 |
| | Mean | 31.21 | 82.79 | 122.69 | 153.55 | 177.42 |
| | 75$^{th}$ Percentile | 42.90 | 97.64 | 139.97 | 172.72 | 198.05 |
| Emerald | 25$^{th}$ Percentile | 10.74 | 42.47 | 64.28 | | |
| shiner | Mean | 19.04 | 50.77 | 75.58 | | |
| | 75$^{th}$ Percentile | 27.35 | 59.08 | 83.89 | | |

*Note:* The means follow a von Bertalanffy growth model and the 25$^{th}$ and 75$^{th}$ percentiles are based on the beta distribution for each age category.

### Population Simulations

The results of the 500 simulations of each 50-year population trajectory were summarized by four plots for each species and pool (e.g., sauger in Robert Byrd pool, Figs. 3 and 4). The first and second plots show results of simulations for the population under the influence of the power plants and the results with the power plant effects removed (i.e., no fish lost via entrainment or impingement or the no-power-plant-effects model). In these plots, the broad, solid line shows the mean of the 500 simulations for each of the 50 years. The dashed lines show the $5^{th}$ and $95^{th}$ percentiles of the 500 simulations for each year. The narrow, solid lines show the minimum and maximum of the simulated populations for each year.

The third plot (Fig. 4) is an overlay of the first two plots and illustrates how the population is predicted to respond if no fish were lost via impingement or entrainment. In these plots, the broad, dashed line is the estimate of the mean population if entrainment and impingement were eliminated (no-power-plants model). The broad, solid line is the estimated population under current conditions with the power plants operating and fish being lost due to impingement and entrainment. If the solid and dashed, broad lines follow a similar path (e.g., bluegill in Pike Island pool, Fig. 5), then no population level impacts would be predicted. However, if these two lines follow noticeably different trajectories (e.g., freshwater drum in Robert Byrd pool, Fig. 6), then impacts to that species in that pool may be occurring. The narrow, dashed line is the $5^{th}$ percentile of the no-power-plants model and the narrow, solid line is the $5^{th}$ percentile for the power plant model.

To help with the interpretation of these boundaries, a fourth plot was developed that illustrates the frequency with which the simulations fall below the $5^{th}$ percentile for more than a specified number of years. For example, for sauger in the Robert Byrd pool (Fig. 4), it can be seen that the probability of falling below the $5^{th}$ percentile for at least one of the 50 years is about 0.55, which is relatively high; the probability of falling below the $5^{th}$ percentile for at least 10 of the 50 years is about 0.06, which is a fairly rare event; and the probability of falling below the 5th percentile for 25 of the 50 years is essentially zero.

The average change for each species and pool that would be expected if power plant entrainment and impingement could be removed is summarized in Table 3. This percent was calculated by finding the geometric mean of the abundance values from the 500 simulations for each year for both the power-plant and no-power-plant simulations. The average expected change for the 50 years is computed from these annual means. The change is calculated by the formula

$$\text{percent change} = 10^{(mn(nopp)\text{ - }mn(pp))} * 100 - 100 \qquad (4)$$

where $mn(nopp)$ is the mean of logarithms of total population of the no power plant simulations and $mn(pp)$ is the mean of logarithms of total population of the power plant simulations.

The projected changes range from a 3% decrease for white bass in Robert Byrd pool to a 57% increase for freshwater drum in Robert Byrd pool when the power plant

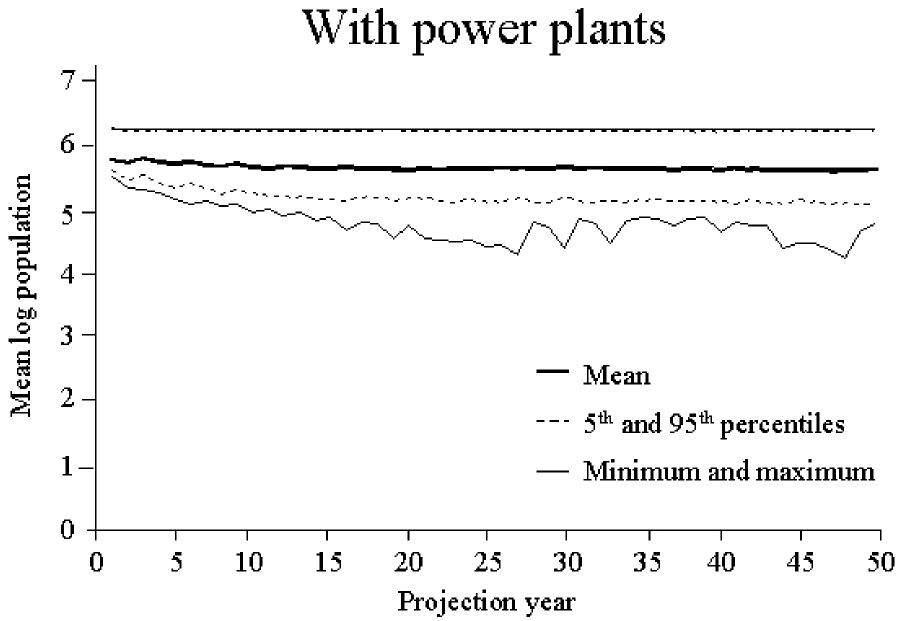

FIGURE 3A

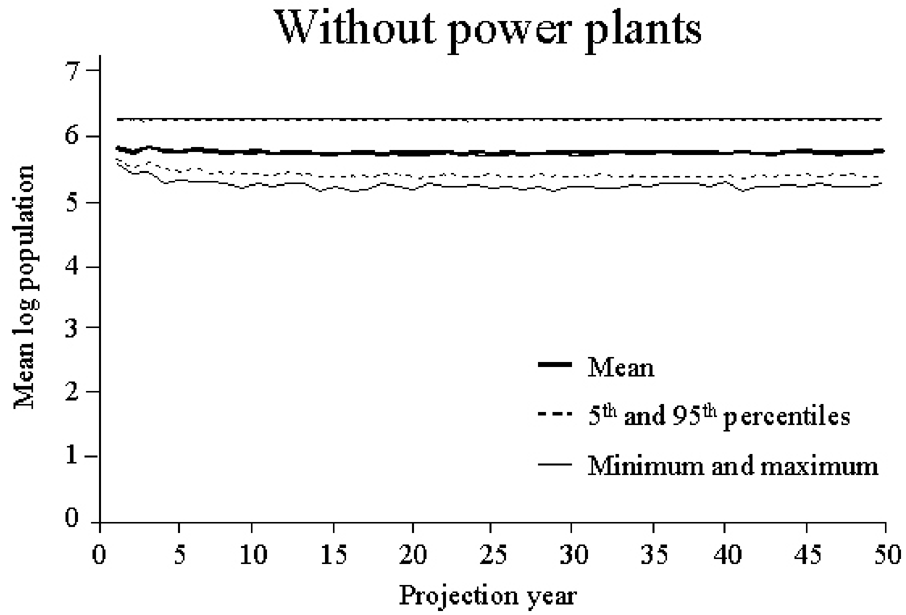

FIGURE 3B

**FIGURE 3.** Simulation results for sauger population in the Robert Byrd pool with and without the influence of power station entrainment and impingement.

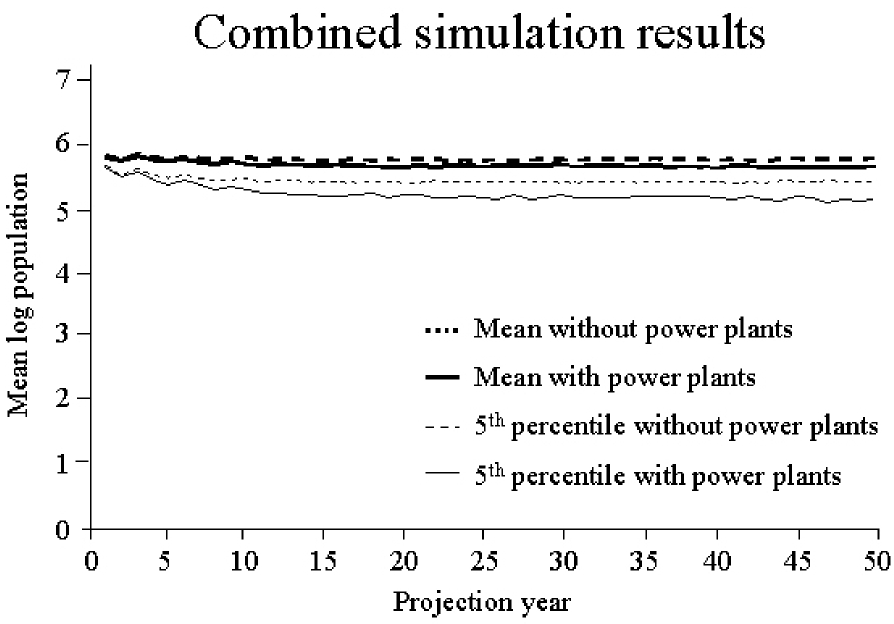

FIGURE 4A

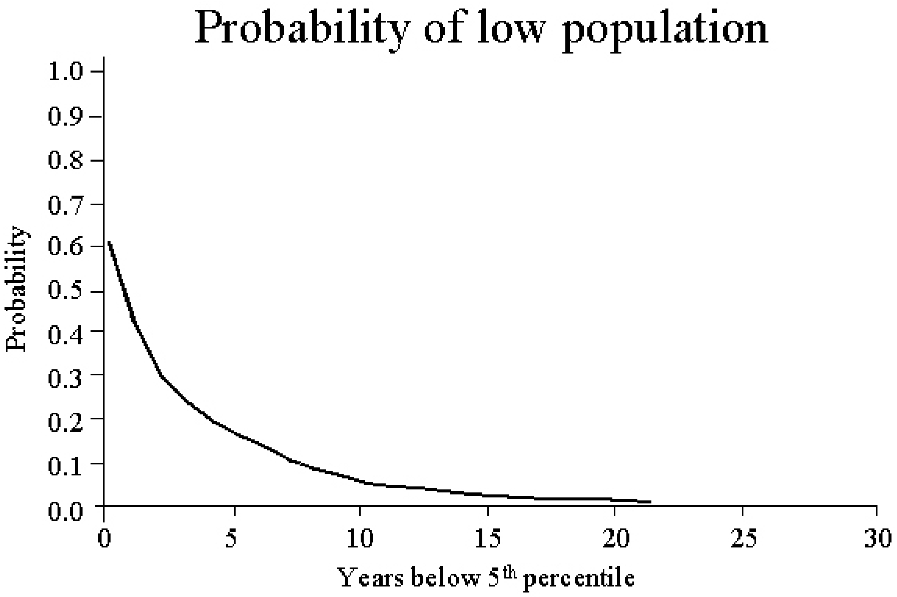

FIGURE 4B

**FIGURE 4.** Combined simulation results for sauger population in the Robert Byrd pool and the probability of falling below the 5th percentile for a specified number of years.

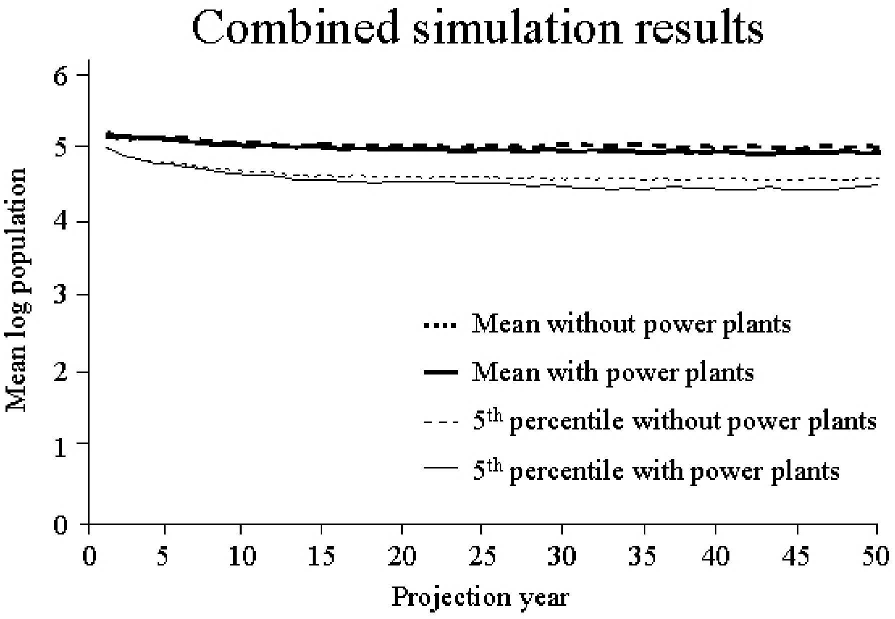

FIGURE 5A

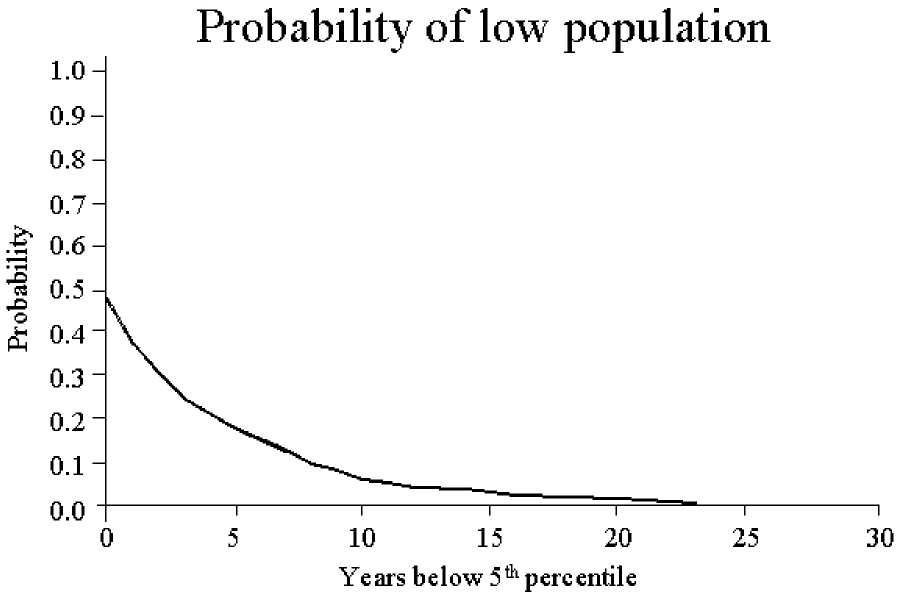

FIGURE 5B

**FIGURE 5.** Combined simulation results for bluegill population in the Pike Island pool and the probability of falling below the 5th percentile for a specified number of years.

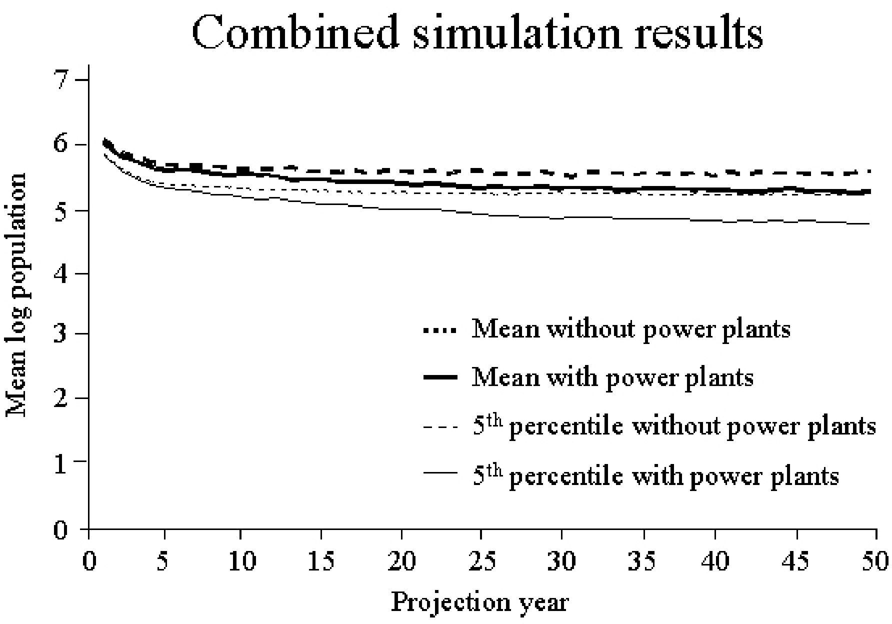

FIGURE 6A

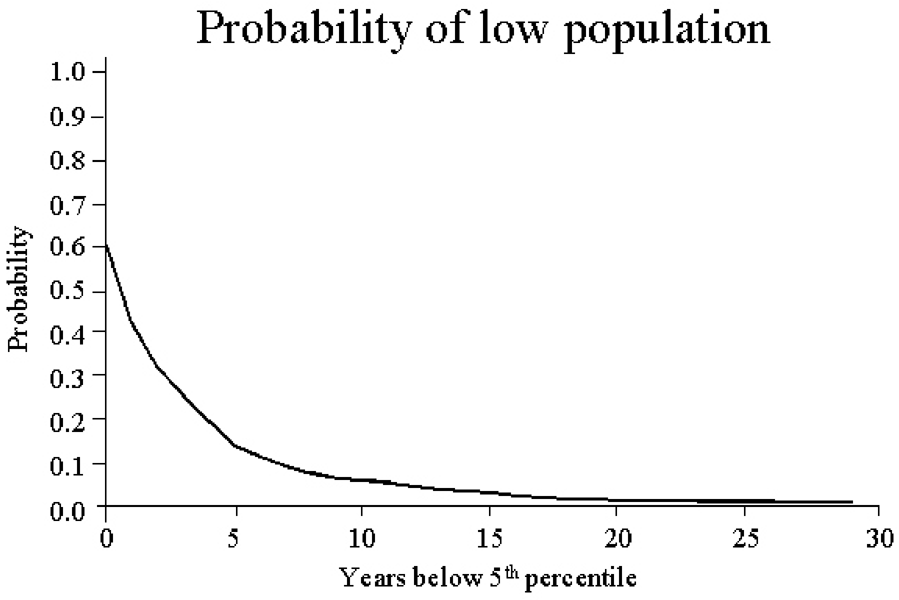

FIGURE 6B

**FIGURE 6.** Combined simulation results for freshwater drum population in the Robert Byrd pool and the probability of falling below the 5th percentile for a specified number of years.

**TABLE 3**
**Estimated Percent Change in the 50-Year Average Population Size of Selected Ohio River Fishes if Impingement and Entrainment Losses are Eliminated through Mathematical Simulation**

| Species | Pool* | Mean Log with Power Plants | Mean Log without Power Plants | Percent Change | Mean** CV (%) | Effect** Level |
|---|---|---|---|---|---|---|
| Sauger | NC | 4.88872 | 4.90693 | 4.28 | 62 | 17.5 |
| | PI | 5.57112 | 5.73589 | 46.14*** | | |
| | RB | 5.60577 | 5.69861 | 23.84*** | | |
| | ML | 6.34186 | 6.34501 | 0.73 | | |
| | MA | 6.03326 | 6.05421 | 4.94 | | |
| Gizzard shad | NC | 6.41621 | 6.53255 | 30.72 | 158 | 44.7 |
| | PI | 7.29137 | 7.29946 | 1.88 | | |
| | RB | 7.06017 | 7.07516 | 3.51 | | |
| | ML | 7.72982 | 7.73173 | 0.44 | | |
| | MA | 6.85997 | 6.85992 | -0.01 | | |
| Freshwater drum | PI | 5.70266 | 5.75293 | 12.27 | 101 | 28.6 |
| | RB | 5.37173 | 5.56635 | 56.54*** | | |
| | ML | 6.08379 | 6.11108 | 6.48 | | |
| | MA | 5.94638 | 6.04799 | 26.36 | | |
| White bass | PI | 5.05217 | 5.07317 | 4.95 | 98 | 27.7 |
| | RB | 5.27650 | 5.26525 | -2.56 | | |
| Bluegill | NC | 5.13175 | 5.15873 | 6.41 | 59 | 16.7 |
| | PI | 4.96286 | 5.01323 | 12.30 | | |
| | RB | 4.93745 | 4.96330 | 6.13 | | |
| | ML | 5.79204 | 5.89857 | 27.80*** | | |
| | MA | 5.29899 | 5.38488 | 21.87*** | | |
| Emerald shiner | PI | 6.44737 | 6.49330 | 11.16*** | 28 | 7.9 |

*NC = New Cumberland pool; PI = Pike Island pool; RB = Robert Byrd pool (formerly Gallipolis Pool); ML = Markland pool; MA = McAlpine pool.
**For comparison, the mean CV is an estimate of typical interannual percent deviation estimated from the observed data record. The effect level is an estimate of the 95$^{th}$ percentile of the percent deviation in the 50-year run. A change larger than this is unlikely to occur as a result of natural variation.
***Projected percent change greater than would be expected from normal year-to-year population variation.

effect is removed. The slight decreases predicted for gizzard shad and white bass are the result of random components of the model resulting in a larger population for the power plant simulations than for the no-power-plant simulations. Because the no-power-plant simulations add impingement and entrainment losses back into the populations, logic dictates that without random effects, the no-power-plant population should always be as large or larger.

## DISCUSSION

### Model Accuracy and Resolution

Despite the lack of site-specific data for some parameters (e.g., fecundity), the population model developed herein provides reasonable guidance as to which populations would most likely increase if entrainment and impingement losses were to be removed. To reduce the possibility of inappropriate decisions being made, a conservative approach was taken at each decision point in compiling the model. Until the model can be refined using additional site-specific data, interpretation of the model outputs should be done cautiously and should not focus on the absolute values for average expected change for a given species and pool. Rather, the focus should be on relative differences among species and pools in the values for average expected change. The resolving power of the model is currently limited by the lack of site-specific age/growth and fecundity data, as well as the inability of electrofishing to capture all age (size) classes equally. Even though an adjustment was made for electrofishing size-dependent efficiency, other unquantifiable factors undoubtedly also affect electrofishing results. For example, larger individuals of species such as sauger and freshwater drum are less shoreline oriented than the smaller individuals of these same species. Thus, the apparent absence of large sauger and freshwater drum in the electrofishing database is likely an artifact associated with the behavioral characteristics of these species. Examination of ORSANCO lock chamber rotenone data for sauger and channel catfish confirmed that larger individuals of these species were more abundant than the electrofishing data suggested[22].

### Impact Predictions

With the above cautions in mind, the likelihood that populations might measurably increase in abundance if entrainment and impingement losses were eliminated was estimated by comparing the predicted percentage increase with the observed population variability shown by each species over the years of the electrofishing survey (Table 3). The interannual population CV was computed for each species and pool, and these estimates were averaged across pools for each species to estimate the typical year-to-year percent variation as it occurs in nature. The effect level was computed to correspond to a level of deviation from normal population levels that would be exceeded in only 5% of cases in a 50-year duration. The 50-year duration was chosen based on the life expectancy of an Ohio River power generating plant. The effect level was computed as (2∗CV/sqrt(50)). When the model predicted a change greater than the effect level, it indicates that the projected population change exceeds what might be reasonably expected due to normal interannual variation.

Overall, the modeling results suggest that improvements at the population level are unlikely in 16 of the 22 cases (Table 4). In 9 of these 16 cases, the increases predicted by the model are small (<5%) and in all 16 cases the predicted increase was

**TABLE 4**
**Likelihood of Impacts to Fish Populations after 50 Years of Entrainment/Impingement Losses based on Leslie Matrix Model Results***

| | River Pool** | | | | |
|---|---|---|---|---|---|
| **Species** | **NC** | **PI** | **RB** | **ML** | **MA** |
| Bluegill | No | No | No | Yes | Yes |
| Freshwater drum | — | No | Yes | No | No |
| Emerald shiner | — | Yes | — | — | — |
| Gizzard shad | No | No | No | No | No |
| Sauger | No | Yes | Yes | No | No |
| White bass | — | No | No | — | — |

*No = No impact expected; Yes = impact may occur; Dash(—) = data insufficient to make comparisons.
**NC = New Cumberland; PI = Pike Island; RB = Robert Byrd (formerly Gallipolis); ML = Markland; MA = McAlpine.

within the level of normal variability expected for populations of these species. In the other 6 of the 22 cases (bluegill in the Markland and McAlpine pools, drum in the Robert Byrd pool, emerald shiner in the Pike Island pool, and sauger in the Pike Island and Robert Byrd pools) the predicted increases (11–57%) were outside the range of expected variability. It is interesting to note that gizzard shad, despite being one of the most abundant fish species in the Ohio River[23] and dominating impingement catches at many power plants[24], is not predicted to increase in any of the pools if power plant entrainment and impingement were removed.

Although the increases predicted in these six cases are statistically real, there is no guarantee that these increases would actually occur, because the changes predicted by the model assume that populations of these species change only in response to entrainment and impingement losses and that none of the entrained or impinged fish survive. In reality, each population will respond to a combination of abiotic (e.g., river flow, water quality, habitat quality) and biotic factors (e.g., predation, exotic species, competition, etc.). In particular, spawning success and year class strength in fishes vary widely, depending on largely unknown interactions of these and other biotic and abiotic factors. Thus, it seems likely that the populations of these species 50 years from now are more likely to be affected by changes in factors such as habitat, water quality, and the occurrence of floods, droughts, and temperature extremes, rather than the minor (typically <10%) changes resulting from entrainment/impingement losses predicted by the model.

### ACKNOWLEDGMENTS

Funding for this study was provided by American Electric Power, American Municipal Power, Buckeye Power, Cinergy, First Energy, Indiana-Kentucky Electric Corp., Ohio Valley Electric Corp., and Vectren. The authors thank Webb Van Winkle, Charles Coutant, and Gary Hickman for providing invaluable comments on an earlier draft of this manuscript.
